# Contemporary Physicians of Texas

**Published:** 1889-05

**Authors:** 


### BIOGRAPHY CONTEMPORARY PHYSICIANS OF TEXAS.

The Texas Biographical Publishing Company, Dr. J. J. Tobin, President, Dr. F. E. Daniel, Secretary and Editor, announce that this great work begun by Dr. Daniel in 1885, and suspended on account of the drought aud hard times, will be pushed to speedy completion, and published this fall. Mr. L. E. Daniell, the well known author of "Personnel of the Texas State Government," is the general agent for this company, and is in the field taking subscriptions. The biography has been secured, as well as the portrait, of most of the Ex-Presidents of the Texas State Medical Association, and a large number of other leading and representative physicians, and it is only necessary now to secure a few more subscribers to complete the list—only twenty more portraits can be accommodated—there being thirty already accepted.

The terms are ten dollars per copy, containing the "write up" or biography of the subscriber. For insertion of portrait—a moss type, fully equal to a steel engraving, $25 extra. No money will be required until the manuscript is ready for the press. As fast as data is sent in, the sketches wiil be prepared; so that by the time the canvass is completed the manuscript will be ready to print, when notice to that effect will be sent to all subscribers, attested by a committee of well known physicians of Austin, who have kindly offered to act as trustees for the company. Two notes of $5 each will be taken from each subscriber, both payable at bank in Austin; the first, on completion of manuscript for the press, and the other on delivery of the book.

The work will be gotten out in first class style, on heavy calendered book paper, and bound in pressed Turkey Morocco and cloth and gilt, making it an ornament to any library or parlor. The engravings, fifty in number, will be executed in the highest style of art. The remaining few subjects will be selected with great care, so as to represent the active element of Texas practitioners only.

It is not alone the "distinguished" of the profession whose biography will appear, but the rank and file of reputable, representative practitioners.

Future generations will enquire who and what were the men who, finding medicine in Texas a chaos, shaped its destiny, advanced its dignity, and placed it in the foremost ranks of science? Who redeemed "Texas Medicine" from the obloquy and obscurity it occupied almost naturally, from the rapid formation of a new State, the pouring in of a heterogeneous population from all quarters of the globe? Who organized it, and separated the wheat from the chaff? drawing a sharp distinction between the representatives of rational Hippocratic medicine and the ephemeral and flimsy "pathies," and the arrant pretenders who flocked to these hospitable shores.

Don't say, therefore, doctor, when you are honored by our agents with a request to enter your biography as that of one of the representative practitioners of Texas: "I have no record; I have done nothing." Every man has a record, all have a future, and it is with you to make a career. When you have done so, let the *data* of your early life be available to the future biographer, who will write you up after you have become "distinguished." What this work aims at, is to put on permanent record the lives of those who are now shaping the destinies of rational medicine in Texas, as well as those who have brought it to its present status.

